# Chimeric Hepatitis B core antigen virus-like particles displaying the envelope domain III of dengue virus type 2

**DOI:** 10.1186/1477-3155-10-30

**Published:** 2012-07-13

**Authors:** Upasana Arora, Poornima Tyagi, Sathyamangalam Swaminathan, Navin Khanna

**Affiliations:** 1 Recombinant Gene Products Group, International Centre for Genetic Engineering & Biotechnology, Aruna Asaf Ali Marg, New Delhi, 110067, India

## Abstract

**Background:**

Dengue is a global public health problem for which no drug or vaccine is available. Currently, there is increasing interest in developing non-replicating dengue vaccines based on a discrete antigenic domain of the major structural protein of dengue viruses (DENVs), known as envelope domain III (EDIII). The use of bio-nanoparticles consisting of recombinant viral structural polypeptides, better known as virus-like particles (VLPs), has emerged as a potential platform technology for vaccine development. This work explores the feasibility of developing nanoparticles based on *E. coli-*expressed recombinant Hepatitis B virus core antigen (HBcAg) designed to display EDIII moiety of DENV on the surface.

**Findings:**

We designed a synthetic gene construct encoding HBcAg containing an EDIII insert in its c/e1 loop. The fusion antigen HBcAg-EDIII-2 was expressed in *E. coli*, purified to near homogeneity using Ni^+2^ affinity chromatography and demonstrated to assemble into discrete 35–40 nm VLPs by electron microscopy. Competitive ELISA analyses showed that the EDIII-2 moieties of the VLPs are accessible to anti-EDIII-2-specific monoclonal and polyclonal antibodies, suggesting that they are surface-displayed. The VLPs were highly immunogenic eliciting high titer anti-EDIII-2 antibodies that were able to recognize, bind and neutralize infectious DENV based on ELISA, immunofluorescence and virus-neutralization assays.

**Conclusion:**

This work demonstrates that HBcAg-derived nanoparticles can serve as a useful platform for the display of DENV EDIII. The EDIII-displaying nanoparticles may have potential applications in diagnostics/vaccines for dengue.

## Findings

Dengue is a mosquito-borne viral disease prevalent in more than a hundred tropical and sub-tropical countries placing about 2.5 billion of the global population at risk, causing 50–100 million infections and ~12,500 deaths each year [1]. There are four serotypes of dengue viruses (DENV-1, -2, -3 and −4), belonging to the family *Flaviviridae*[2], each of which can cause dengue disease [1]. The tremendous challenges being faced in the development of live viral dengue vaccines [3] has spurred a keen interest in new generation non-replicating subunit vaccines [3,4]. In this context, a discrete domain of the viral envelope protein has been identified as a potential subunit vaccine candidate [3-5]. This domain known as envelope domain III (EDIII), is exposed and accessible on the virion surface [6], contains multiple type- and subtype-specific neutralizing epitopes [7] and is implicated in host receptor binding [8].

One way to augment the vaccine potential of EDIII would be to display it in multiple copies on the surface of a nanoparticle carrier. Naturally occurring bio-nanomaterials, by virtue of their biocompatibility and biodegradability, are emerging as preferred platforms for biomedical applications [9]. Viruses are naturally occurring nanoparticles whose particulate nature with repetitive coat protein architecture and pathogen associated molecular patterns make them a potentially valuable platform for vaccine development. A subclass of viral nanoparticles can be generated in heterologous expression systems exploiting the propensity of several recombinant viral coat proteins to self-assemble. These genome-free viral nanoparticles which eliminate the infectious biohazard inherent in the naturally occurring viral nanoparticles are more popularly known as virus-like particles (VLPs) [9,10]. The 183 amino acid (aa) residue Hepatitis B virus core antigen (HBcAg) which can assemble into VLPs is a well-documented carrier of foreign antigens from several different pathogens [11] and HBcAg-based VLP vaccine candidates for malaria and influenza are being evaluated in clinical trials [12,13]. Of relevance to our study is the reported capacity of HBcAg to accommodate foreign inserts (in the size range of 100–260 aa residues) in its surface-exposed c/e1 loop, with retention of its VLP-forming ability. This loop which contains the major B-cell epitopes ‘c’ and ‘e1’ is also known as the major immunodominant region of the HBcAg VLPs [11]. Optimal immunogenicity of the foreign insert is ensured by the concomitant removal of these major HBcAg immunodominant epitopes. Interestingly, many of these chimeric VLPs have been produced using *E. coli* expression hosts [14-20]. In this study, we report the design, creation and characterization of chimeric nanoparticles containing DENV-2 EDIII (herein indicated as EDIII-2) inserted into the c/e1 loop of HBcAg. Further we show that these chimeric VLPs display EDIII-2 on the surface and elicit antibodies specific to DENV-2.

**Figure 1:**
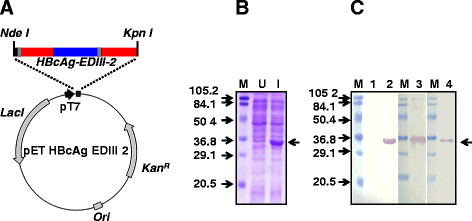
**Design and expression of HBcAg-EDIII-2 antigen in*E. coli*.** (**A**) A map of the HBcAg-EDIII-2 expression vector. The synthetic *HBcAg-EDIII-2* gene is inserted under the control of the phage T7 promoter (pT7) in pET29a. The organization of different segments of this fusion gene is indicated in colour as follows. The HBcAg- and EDIII-2-encoding regions are shown in red and blue, respectively. The 6x His tag-encoding sequences at the 5’end is shown in black. The two linker-encoding sequences, the first following the 6x His tag and the second after the EDIII-2 encoding sequences, are shown in grey. Other abbreviations are as follows. *Lac I*: *Lac* repressor gene; *Kan*^*R*^: Kanamycin marker; *Ori*: Replication origin sequences. (**B**) SDS-PAGE analysis of recombinant HBcAg-EDIII-2 expression. This panel displays the Coomassie-stained polypeptide profiles of lysates prepared from un-induced (U) and induced (I) *E. coli* cells harboring the plasmid shown in A. Pre-stained protein molecular weight markers were run in lane ‘M’. Their sizes (in kDa) are shown at the left of the panel. The arrow on the right indicates the position of the recombinant HBcAg-EDIII-2 protein. (**C**) Immunoblot analyses of recombinant protein expression. Aliquots of un-induced and induced cell lysates (described in panel ‘B’) were electrophoresced, electroblotted onto nitrocellulose membranes and probed with anti-EDIII mAb 24A12 (lane 2), penta His mAb (lane 3), or anti-HBcAg mAb ab8638 (lane 4). An aliquot of the un-induced cell lysate was probed with mAb 24A12 (lane 1). Pre-stained protein size markers were run in lanes marked ‘M’. Their sizes (in kDa) are indicated to the left of the first blot. The arrow to the right indicates the position of the recombinant HBcAg-EDIII-2 protein.

The chimeric HBcAg-EDIII-2 antigen was designed by replacing aa residues 76–80 in the c/e1 loop of a C-terminally truncated HBcAg molecule (lacking aa residues 166 to 183) with the 104 aa residue EDIII-2. We introduced a spacer (GSGDEGG) between the C-terminus of the EDIII-2 insert and aa 81 of HBcAg to minimize any disruption of particle assembly through potential interactions between β-sheet forming residues of EDIII-2 and aa 80–90 of HBcAg [21]. To aid in purification the chimeric antigen design included an N-terminal 6x His tag linked through a pentaglycyl spacer to the N-terminal end of HBcAg. A synthetic gene, *HBcAg-EDIII-2*, encoding this chimeric fusion antigen (GenBank accession # JQ729976), codon-optimized for *E. coli* expression, was inserted into an IPTG-inducible prokaryotic expression vector (Figure 1A and Additional file 1: Figure S1). *E. coli* transformed with this plasmid, expressed the fusion antigen upon induction, (Figure 1B). The identity of this induced protein band was confirmed using antibodies specific to each of the two fusion partners as well as to the affinity tag by immunoblotting analyses (Figure 1C).

**Figure 2:**
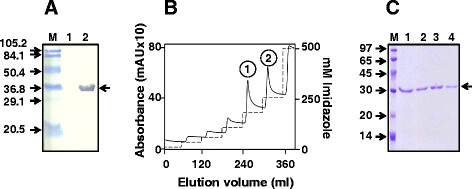
**Affinity purification of the recombinant HBcAg-EDIII-2 protein under denaturing conditions.** (**A**) Western blot analysis of localization of HBcAg-EDIII-2 expression. Induced cells were sonicated and centrifuged. The resultant supernatant (lane 1) and pellet (lane 2) fractions were boiled in Laemmli loading buffer, electrophoresced under denaturing conditions and subjected to immunoblot analysis using mAb24A12 to identify the recombinant HBcAg-EDIII-2 protein. (**B**) Ni^2+^-affinity purification of HBcAg-EDIII-2 from induced *E. coli* cells. The insoluble pellet obtained after sonication of induced cells was purified using Ni^2+^-Sepharose under denaturing conditions. The solid curve represents the chromatographic profile obtained by measurement of absorbance at 280 nm. The two peaks discernible in the elution profile are numbered 1 and 2. The dotted curve represents the imidazole step-gradient employed for elution. (**C**) SDS-PAGE analysis of fractions corresponding to peaks 1 (lanes 1 & 2) and 2 (lanes 3 & 4) shown in panel ‘B’. Low molecular weight protein markers were run in lane ‘M’; their sizes (in kDa) are indicated to the left of the panel. The arrow at the right of the panels A and C indicates the position of the HBcAg-EDIII-2 protein.

**Figure 3:**
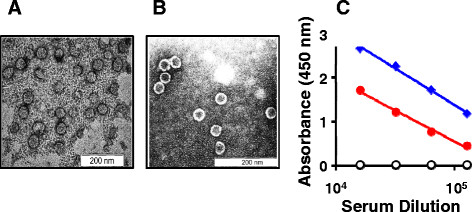
**Characterization of the purified HBcAg-EDIII-2 antigen.** (**A**) The panel depicts VLPs formed by purified HBcAg-EDIII-2 protein (expressed in *P.pastoris*). Peaks 1 and 2 (shown in Figure 2B) were pooled, dialyzed, stained with 1% uranyl acetate and visualized under the electron microscope. (**B**) Electron microscopic visualization of the purified HBcAg carrier (*expressed in E. coli*). (**C**) Evaluation of the immunogenicity of the HBcAg-EDIII-2 antigen by ELISA. Antisera from mice immunized with the fusion antigen (solid red circles) and its precursors (HBcAg: empty black circles; EDIII-2: solid blue diamonds) were analyzed in an ELISA using recombinant EDIII-2 antigen (produced using *P. pastoris*) as the coating antigen.

A localization analysis of the induced cell lysate revealed the fusion antigen to be associated exclusively with the insoluble fraction (Figure 2A). This is consistent with the behavior of a multitude of heterologous proteins over-expressed in *E. coli*. Attempts to obtain the fusion antigen in the soluble phase by performing the IPTG induction at lower temperatures failed. While induction at 16 °C did not result in discernible expression of the recombinant antigen, at 25 °C it was barely discernible. Expression was evident at higher induction temperatures ( Additional file 1: Figure S2), but the expressed protein tended to be associated with the insoluble fraction. Consequently, we purified the protein from induced cells under denaturing conditions (Figure 2B). An imidazole step-gradient elution resulted in the emergence of two protein peaks, 1 and 2. Interestingly, an SDS-PAGE analysis of the two peaks revealed them to contain a single polypeptide co-migrating with the ~30 kDa low molecular weight marker. This mobility is consistent with the predicted size (~31 kDa) of the HBcAg-EDIII-2 antigen. As both peaks in Figure 2B appeared to be similar (Figure 2C), we pooled the two and dialyzed the material (0.3 mg/ml) against 25 mM sodium bicarbonate buffer, pH 9.2. We could recover ~95% of the protein after dialysis. Analysis of an aliquot of this by electron microscopy revealed the presence of discrete VLPs of ~35-40 nm (Figure 3A). Interestingly, these were quite similar to purified, *E. coli*-expressed VLPs (Figure 3B). It is noteworthy that the HBcAg-EDIII-2 protein had been purified under highly denaturing conditions. Yet, the dialyzed protein could be observed to be organized into VLPs. This underscores the fact that inherent propensity of HBcAg to self-assemble into VLPs is not compromised by the insertion of EDIII-2 into its c/e1 loop. Starting from a liter of induced *E. coli* culture we obtained ~7 mg of HBcAg-EDIII-2 VLPs ( Additional file 1: Table S1).

**Figure 4:**
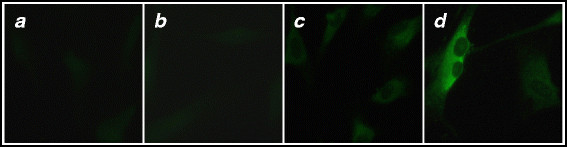
**Evaluation of antibodies elicited by HBcAg-EDIII-2 fusion antigen by indirect immunofluorescence assay.** Sera from mice that were mock-immunized (panel *a*), and immunized with HBcAg (panel *b*), EDIII-2 (panel *c*) or HBcAg-EDIII-2 (panel *d*) antigens were used as the primary antibody to pick up DENV-2 in infected BHK-21 cells, in conjunction with an anti-mouse IgG-FITC conjugate.

Next, we examined if these VLPs display the EDIII-2 to the immune system and induce specific antibodies. To this end, we immunized Balb/C mice with these VLPs and tested the immune sera for anti-EDIII-2 antibodies in an indirect ELISA using purified EDIII-2 [22] as the coating antigen. In parallel, we also tested sera from mice immunized with purified EDIII-2 and HBcAg antigens. The data in Figure 3C reveal that the fusion antigen does indeed elicit high titers of anti-EDIII-2 antibodies, lending support to the notion that the chimeric VLPs do indeed facilitate display of EDIII-2 efficiently to the immune system. However, the anti-EDIII-2 antibody titers elicited by the fusion antigen appeared to be slightly but consistently lower than those elicited by the EDIII-2 antigen. This we believe could be a reflection of the ~3 fold higher concentration of EDIII-2 antigen compared to the HBcAg-EDIII-2 fusion antigen, per immunization dose, as the latter is ~3 times larger in size compared to the former. This leads to the suggestion that the anti-EDIII-2 antibody titers elicited by the two antigens may indeed be quite comparable. The next question we addressed was, would these antibodies also recognize and bind to infectious DENV-2. For this, we infected BHK cells with DENV-2 and probed them with anti-HBcAg-EDIII-2 antiserum, in conjunction with a secondary antibody FITC conjugate. The indirect immunofluorescence data in Figure 4 show that the antibodies elicited by the fusion antigen were as good as those elicited by EDIII-2, in picking up DENV-2 in infected BHK cells. We next performed a virus neutralization assay to determine the titers of DENV-2 virus-neutralizing antibody titers in these antisera using plaque reduction neutralization test (PRNT) as described previously [23]. The PRNT_50_ titers elicited by EDIII-2 and the HBcAg-EDIII-2 fusion antigens were, respectively, 10 and 35.

**Figure 5:**
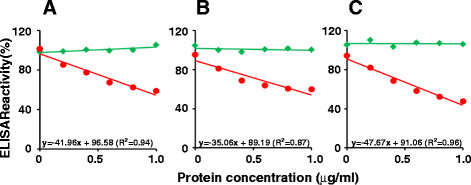
**Competitive ELISA.** Anti-EDIII mAb24A12 (panel A), anti-EDIII-T antiserum (panel B) and anti-HBcAg-EDIII-2 antiserum (panel C) were separately pre-incubated with varying concentrations of purified HBcAg (green curves) and HBcAg-EDIII-2 (red curves) VLPs, and analyzed for residual anti-EDIII-2 antibodies using purified *P. pastoris*-expressed EDIII-2 antigen as the coating antigen. ELISA reactivity in the absence of any added protein in the pre-incubation step was taken to represent 100%. The regression equations for the HBcAg-EDIII-2 competition assays (red curves) are shown just above the horizontal axis in each panel.

The above data suggest that the fusion antigen VLPs display the EDIII-2 moiety as expected. Then, it must be accessible to antigen combining sites on anti-EDIII mAbs and polyclonal antibodies. We tested this using a competitive ELISA approach, the results of which are shown in Figure 5. In this experiment we used three different antibodies, an EDIII-specific mAb 24A12 [22], a murine polyclonal antiserum obtained by immunization with a chimeric antigen containing the EDIIIs of all 4 DENVs linked in a tandem array [24], and the murine polyclonal antiserum raised using the HBcAg-EDIII-2 antigen. Remarkably, in all three cases, we observed that pre-incubation of antibodies with the HBcAg-EDIII-2 antigen, but not with the HBcAg antigen (lacking EDIII-2), resulted in a dose-dependent depletion of antigen-combining sites available to bind EDIII-2 antigen coating the microtiter wells. These observations strongly support the notion that the chimeric VLPs do display the EDIII-2 moiety on the surface in a manner that is compatible with recognizing and binding to specific anti-EDIII-2 antibodies.

In conclusion, this work has demonstrated the feasibility of using an *E. coli* expression system to produce chimeric nanoparticles using a fusion antigen comprising of the HBcAg polypeptide with EDIII-2 inserted into its surface-exposed c/e1 loop. It has shown further that the EDIII-2 moiety is displayed on the surface of the chimeric nanoparticle and is able to induce the production of specific antibodies capable of binding and neutralizing the infectivity of DENV-2. This work provides the basis for us to envisage next generation HBcAg-derived mosaic nanoparticles that display the EDIII domains of not one, but all four DENV serotypes. Such nanoparticles could have potentially interesting and possibly useful diagnostic and vaccine potential.

## Abbreviations

aa, Amino acid; BHK, Baby hamster kidney; DENV, Dengue virus; DENV-2, Dengue virus type 2; EDIII, Envelope domain III, EDIII-2, Envelope domain III of dengue virus type 2; ELISA, Enzyme-linked immunosorbent assay; FITC, Fluorescene isothiocyanate; HBcAg, Hepatitis B virus core antigen; mAb, Monoclonal antibody; SDS-PAGE, Sodium dodecyl sulfate-polyacrylamide gel electrophoresis; VLP, Virus-like particle.

## Competing interests

The authors declare that they have no competing interests.

## Authors’ contributions

SS and NK conceived and designed the study. UA and PT performed experiments. UA, SS and NK prepared the manuscript. All authors read and approved the manuscript.

## Supplementary Material

Additional file 1: **The file is organized into 3 sections.** Section S1 describes essential Methods. Section S2 provides supplementary figures pertaining to the fusion antigen design and sequence (Figure S1) and the effect of induction temperature on HBcAg-EDIII-2 expression (Figure S2). Section S3 provides a summary of HBcAg-EDIII-2 purification (Table S1). Click here for file
