# Supplementary material for: Chimeric Hepatitis B core antigen virus-like particles displaying the envelope domain III of dengue virus type 2
Source: J Nanobiotechnology. 2012 Jul 13;10:30. doi: 10.1186/1477-3155-10-30 (PMC3411447; doi:10.1186/1477-3155-10-30)
Supplement: Additional file 1: — The file is organized into 3 sections. Section S1 describes essential Methods. Section S2 provides supplementary figures pertaining to the fusion antigen design and sequence (Figure S1) and the effect of induction temperature on HBcAg-EDIII-2 expression (Figure S2). Section S3 provides a summary of HBcAg-EDIII-2 purification (Table S1). [file 1477-3155-10-30-S1.doc]

**Additional Data Files**

**Section S1: Methods**

- 1. **Plasmid construction**

Synthetic *HBcAg-EDIII-2* and *HBcAg* genes, codon-optimized for *E. coli* expression, were cloned separately into plasmid pET29a and pET28a, respectively. Kanamycin-resistant transformants were screened by colony PCR and confirmed by restriction analysis. Plasmid construction was carried out in DH5α. For expression, the plasmids were transformed into *E. coli* BL21 (DE3) and induced with IPTG.

- 1. **Purification of recombinant proteins**

One liter culture of logarithmically growing *E. coli* BL21 (DE3) harboring the HBcAg-EDIII-2 expression construct was induced with 1 mM IPTG at 37^o^C for 4 hours. The induced cell pellet (~2 g wet weight) was washed with 50 ml 1x PBS and lysed by sonication in 20 ml 50 mM sodium phosphate, pH 8 containing 0.2% Tween 20. The lysate was spun down (13,000 rpm/1 hour/4^o^C) and the resultant pellet extracted overnight at 4^o^C using 10 ml extraction buffer (6M Guanidine-HCl/0.3M NaCl/50 mM imidazole/0.5% Tween 20/20 mM Tris-HCl, pH 8). The extract was spun down as before and the resultant supernatant fraction bound in batch mode to 5 ml Ni^2+^-Sepharose for 4 hours at room temperature (RT). This was packed into a column and washed with 10 bed volumes of the extraction buffer and eluted with 50-500 mM imidazole step gradient in 8M urea/10 mM Tris-HCl/100 mM NaH_2_PO_4_, pH 8. Fractions were analyzed by SDS-PAGE. Purified fractions were pooled and dialyzed against 25 mM sodium bicarbonate buffer, pH 9.2. Recombinant HBcAg expressed in *E. coli* was purified from the soluble fraction using a previously reported protocol (Yap et al: *J Virol Meth* 2009, **160:** 125-131).

- 1. **Immunological analyses**

Immunizations were done using groups of 4-6 week old Balb/C mice (n=6) in accordance with animal ethics guidelines of the Government of India. There was one control group (mock-immunization) and three antigen-immunization groups (HBcAg, EDIII-2 and HBcAg-EDIII-2). Mice were injected i.p. with 20 μg antigen (in 100 μl volume formulated using 500 μg alum) on days 0, 30 and 90. Sera were collected 1 week after the final immunization and analyzed as follows.

For indirect ELISA, microtiter wells were coated with *P. pastoris*-expressed, purified EDIII-2 antigen overnight at 4^o^C (250 ng/well in 100 μl 100 mM sodium bicarbonate buffer, pH 9.2). Coated wells were washed (1x PBS/0.1% Tween 20) 3x and blocked (5% skim milk/1x PBS/0.1% Tween 20) for 2 hours at 37^o^C. After washing as before, serial dilutions (in 2.5% skim milk/1x PBS/0.1% Tween 20) of antisera from the different immunization groups were added to the wells and allowed to incubate 1 hour at 37^o^C. Wells were washed 6x and incubated with anti-mouse IgG-HRPO conjugate (0.1 μg/ml dilution buffer; 100 μl/well) for 1 hour at 37^o^C. Wells were washed again and incubated with TMB substrate (100 μl/well) for 30 minutes at 37^o^C. Reactions were stopped using 1N H_2_SO_4_ and absorbance read at 450 nm.

Competitive ELISAs were performed using EDIII-specific mAb 24A12 (10 ng/ml), anti-EDIII-T antiserum (1: 10,000 dilution) and anti-HBcAg-EDIII-2 antiserum (1: 10,000 dilution) as the source of the primary antibody. One hundred μl aliquots of these antibodies were pre-incubated (1 hour at 37^o^C) with equal volumes of purified HBcAg or HBcAg-EDIII-2 proteins (in the concentration range 0-8 μg/ml) and then added to EDIII-2-coated microtiter wells (100 μl/well), prepared as above. From this point onwards, the procedure was as described for indirect ELISA.

For Western blot analyses, samples were electrophoresced on SDS-15% polyacrylamide gels, electro-transferred onto nitrocellulose membrane (25 mM Tris/192 mM glycine/ 20% methanol; 12V for 30 minutes), blocked (5% skim milk/1x PBS/0.1% Tween 20) for 2 hours at RT, rinsed once (1x PBS/1% Tween 20) and incubated with EDIII-specific mAb 24A12 (1μg/ml), anti-His mAb (0.1 μg/ml) or anti-HBcAg mAb ab8638 (1μg/ml). Blots were washed and developed using anti-mouse IgG-HRPO conjugate plus TMB substrate.

For immunofluorescence assay, BHK cells were seeded on coverslips and infected with DENV-2 (m.o.i=0.2). At 36 hours post-infection cells were fixed with 4% formaldehyde for 15 minutes and permeabilized with ice-cold methanol for 15 minutes. The coverslips were washed 3x (50mM Tris-HCl, pH 8.5/500 mM NaCl/0.1% Tween 20), blocked (5% PVP/25% goat serum in wash buffer; 2 hours at 37^o^C), washed again and probed with a 1:50 dilution of sera from the four immunization groups described above. Bound anti-DENV-2 antibodies were visualized under a fluorescence microscope using anti-mouse IgG-FITC conjugate (25 μg/ml).

- 1. **Virus neutralization assay**

Neutralizing antibody titers in immune sera were determined using PRNT. DENV-2 (30-40 plaque forming units in 100 μl) was pre-incubated with an equal volume of serial two-fold dilutions of heat-inactivated (56^o^C/30 minutes), pooled serum collected from immunized mice. All dilutions were done using DMEM+2% heat-inactivated fetal calf serum (∆FCS). After 1 hour pre-incubation at 37^o^C, the virus+serum mixtures were used to infect Vero cells seeded a day in advance in 24-well plates. Each serum dilution was assayed in triplicate wells (200 μl/well). The inoculum was aspirated off after 1 hour and cells overlaid with 0.8% methylcellulose in DMEM + 6% ∆FCS (1 ml/well). Appropriate controls were set up in parallel with negative control (mock-infected) wells receiving 200 µl DMEM + 2% ∆FCS and positive control wells receiving DENV-2 pre-incubated with plain DMEM + 2% ∆FCS, instead of murine serum. Four days later viral plaques were developed using a modification of the immunostaining protocol described earlier with a pan-DENV-specific monoclonal antibody, 4G2. The antiserum dilution resulting in 50% reduction in plaque count (with reference to the number of plaques generated by the virus in the absence of antiserum), was expressed as the PRNT_50_ titer.

**Section S2: Figures**


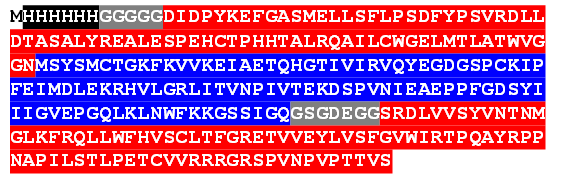

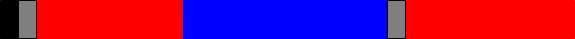


**H L Core ‘n’ (74) EDIII-2 (104) L Core ‘c’ (85)**

**A**

**B**

**Figure S1.** The fusion antigen HBcAg-EDIII-2. (A) Schematic representation of the design of the fusion antigen. The amino and carboxy-terminal halves of HBcAg are denoted by Core ‘n’ and Core ‘c’, respectively. A 6x His tag, (H) is located at the N-terminus of the fusion antigen. The DENV-2 envelope domain III (EDIII-2) is fused into the middle of HBcAg (in the c/e1 loop). There are two different linkers (L), one linking the 6x His tag to Core ‘n’ and the other linking carboxy-terminus of EDIII-2 to Core ‘c’. (B) The amino acid (aa) sequence of the HBcAg-EDIII-2 fusion antigen. The aa sequences are highlighted in colors corresponding to those shown in ‘A’.


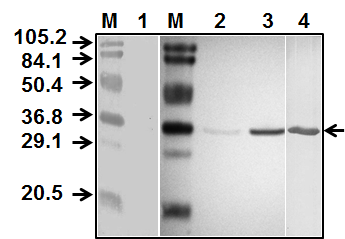


**Figure S2.** Effect of temperature of induction on HBcAg-EDIII-2 expression. *E. coli* cells harboring the pET-HBcAg-EDIII-2 plasmid were induced with 1 mM IPTG at different temperatures, lysed, run on denaturing polyacrylamide gels and electro-transferred to nitrocellulose membrane. The membrane was probed with EDIII-specific mAb24A12 and developed using anti-mouse IgG-HRPO plus TMB substrate. Induction temperatures tested were, 16^o^C (16 hours), 25^o^C (6 hours), 30^o^C (5 hours) and 37^o^C (4 hours) in lanes 1, 2, 3 and 4, respectively. Pre-stained protein markers were run in lanes marked ‘M’. Their sizes (in kDa) are shown to the left. The arrow on the right denotes the position of the HBcAg-EDIII-2 antigen.

**Section S3: Tables**

**Table S1:** Purification of recombinant HBcAg-EDIII-2 antigen from 1L induced *E. coli*

| **Step** | **Total**  **protein (mg)** *^a^* | **Specific**  **protein (mg)***^b^* | **% Purity***^c^* | **%Yield***^d^* |
| --- | --- | --- | --- | --- |
| Gu-HCl extract | 27 | 12 | 44 | 100 |
| Affinity chromatography | 7.0 | 6.6 | 94.3 | 55 |

*^a^*Estimated using BCA method with BSA as reference

*^b^*Determined using Ni-NTA His-Sorb ELISA with His-tagged EDIII-2 as reference

*^c^* % Purity=(Specific protein/Total protein) x100

*^d^*With reference to specific protein content in Gu-HCl extract taken as 100%
